# Supplementary material for: Fatty Hepatocytes Induce Skeletal Muscle Atrophy In Vitro: A New 3D Platform to Study the Protective Effect of Albumin in Non-Alcoholic Fatty Liver
Source: Biomedicines. 2022 Apr 21;10(5):958. doi: 10.3390/biomedicines10050958 (PMC9139027; doi:10.3390/biomedicines10050958)
Supplement: Supplementary file 1 [file biomedicines-10-00958-s001.zip › Supplementary materials.pdf]

biomedicines

MDPI

Article

Fatty Hepatocytes Induce Skeletal Muscle Atrophy In Vitro: A New 3D Platform to Study the Protective Effect of Albumin in Non-Alcoholic Fatty Liver

Francesco De Chiara [1,*,†], Ainhoa Ferret-Miñana [1,†], Juan M. Fernández-Costa [1], Alice Senni [1], Rajiv Jalan [2] and Javier Ramón-Azcón [1,3]

[1] Biosensors for Bioengineering Group, Institute for Bioengineering of Catalonia (IBEC), The Barcelona Institute of Science and Technology (BIST), Baldiri I Reixac 10-12, 08028 Barcelona, Spain; aferret@ibecbarcelona.eu (A.F.-M.); jfernandez@ibecbarcelona.eu (J.M.F.-C.); alice.senni@outlook.it (A.S.); jramon@ibecbarcelona.eu (J.R.-A.)
[2] UCL Institute of Liver and Digestive Health, University College London, London NW3 2QG, UK; rjalan@ucl.ac.uk
[3] ICREA-Institució Catalana de Recerca i Estudis Avançats, 08010 Barcelona, Spain
* Correspondence: fdechiara@ibecbarcelona.eu
† These authors contributed equally to this work.

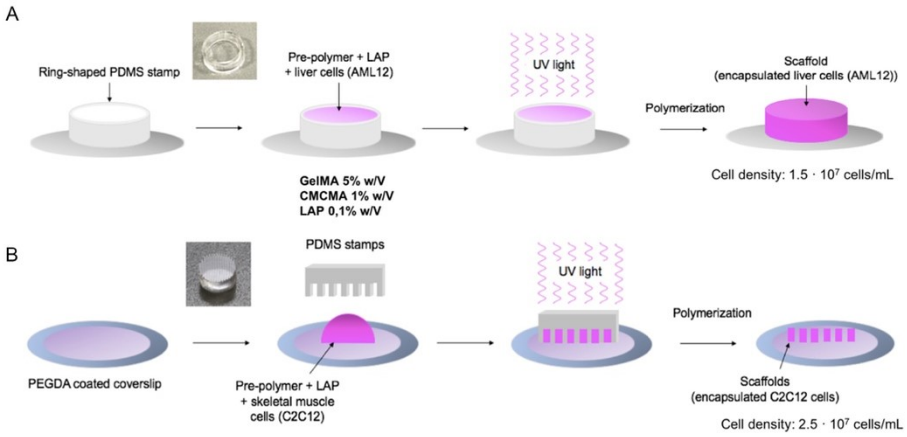

Supplementary Figure S1. Schematic representation of the (A) hepatocytes and (B) myoblasts encapsulation. GelMA: gelatin methacryloyl; CMCMA: carboxymethyl cellulose methacrylate; UV: ultraviolet; PDMS: polydimethylsiloxane; LAP: lithium phenyl(2,4,6-trimethylbenzoyl)phosphonate.

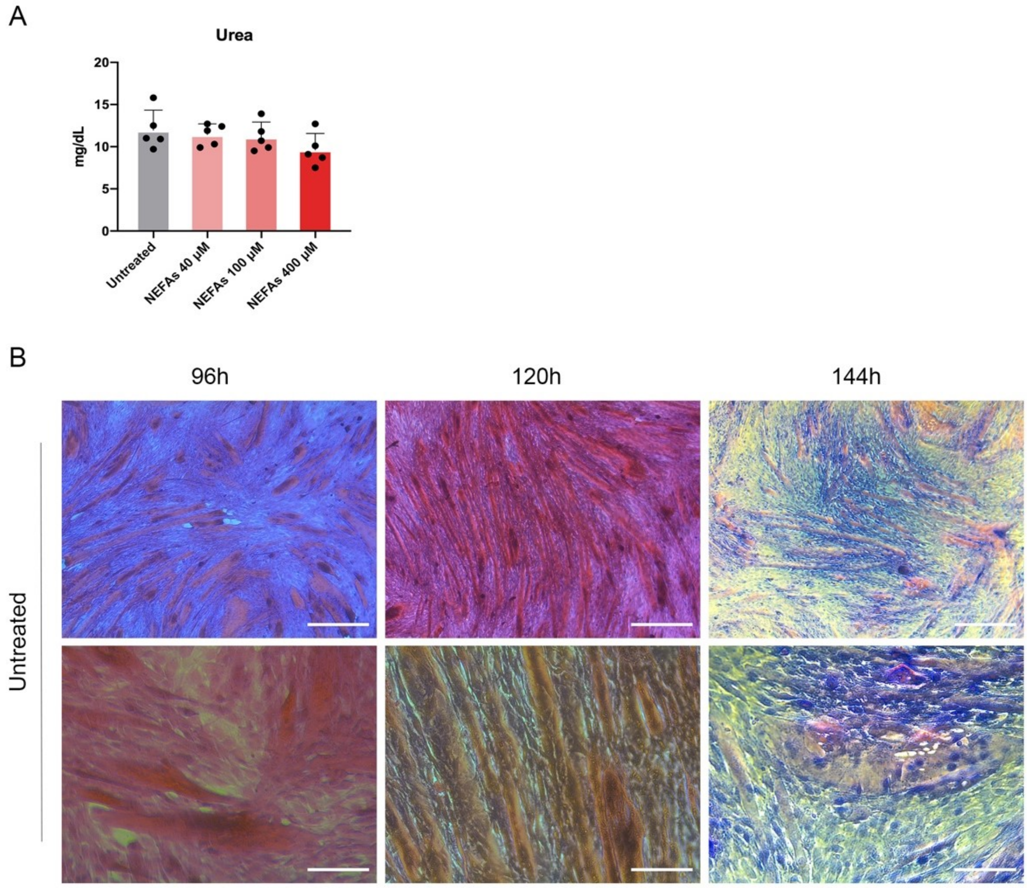

Supplementary Figure S2. (A) Biochemical analyses of the urea from the supernatant of AML12 challenged with NEFAs for 72 hours assessed by clinical standard procedures. (B) Hematoxylin and eosin staining of untreated C2C12 cells at 96, 120 and 144 hours after culture. Scale bar top panel = 400 µm, and bottom panel = 100 µm.

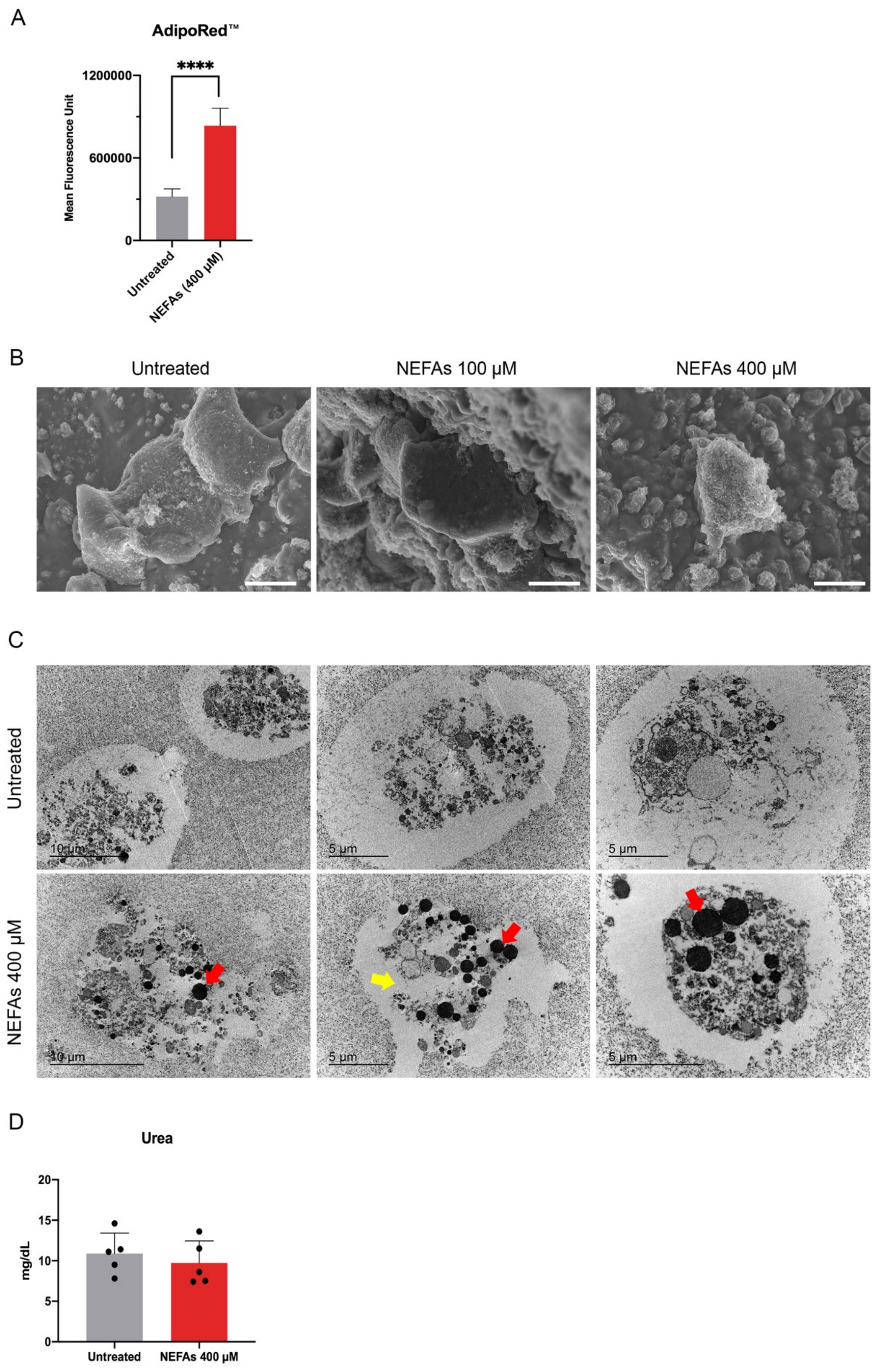
A
AdipoRed™
****
1200000
600000
0
Mean Fluorescence Unit
Untreated
NEFAs (400 µM)
B
Untreated
NEFAs 100 µM
NEFAs 400 µM
C
Untreated
NEFAs 400 µM
10 µm
5 µm
5 µm
10 µm
5 µm
5 µm
D
Urea
20
15
10
5
0
mg/dL
Untreated
NEFAs 400 µM

Supplementary Figure S3. (A) Quantification of the lipids accumulation in NEFAs treated hepatocytes assessed by AdipoRed™ assay. (B) Ultrastructural assessment of untreated and treated (NEFAs 100 μM and 400 μM) AML12 cells phenotype by scanning electron microscopy. Scale bar = 10 μm. (C) Ultrastructural assessment of untreated and treated (NEFAs 100 μM and 400 μM) AML12 cells phenotype by transmission electron microscopy. Red and yellow arrows indicate intracellular lipid accumulation and cytoplasmatic membrane disruption, respectively. (D) Biochemical analyses of the urea from the supernatant of 3D AML12 challenged with NEFAs for 72 hours assessed by clinical standard procedures. The results are expressed as mean values ± SEM and compared using one-way analysis of variance followed by post hoc tests when appropriate. **** p<0.0001.

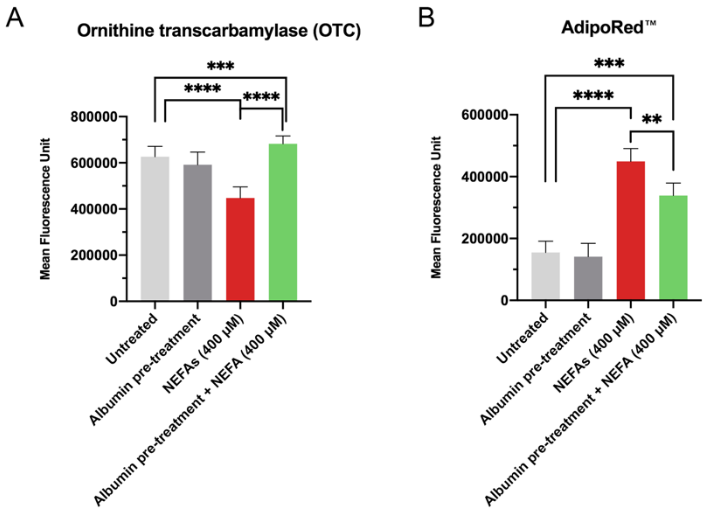

Supplementary Figure S4. (A) Quantification of the ornithine transcarbamylase (hepatocyte's functionality marker) of 3D AML12 cells pre-treated with albumin and challenged with NEFAs for 72 hours obtained from 5 random z-Stacks/condition using immunostaining. (B) Quantification of lipids accumulation of 3D AML12 cells pre-treated with albumin and challenged with NEFAs for 72 hours obtained from 5 random z-Stacks/condition assessed by AdipoRed™ assay. The results are expressed as mean values ± SEM and compared using one-way analysis of variance followed by post hoc tests when appropriate. ** p<0.01; *** p<0.001; **** p<0.0001.

Supplementary Video S1. Brightfield microscopy video of 2D AML12 cells challenged with NEFAs 400 μM for 24 hours.

Supplementary Videos S2 and S3. Confocal images of live/dead assay of encapsulated AML12 cells, 50 and 150 μL, respectively.

Supplementary Video S4. Z-stack reconstruction of lipids accumulation in NEFAs treated hepatocytes assessed by AdipoRed™ assay.

Supplementary Table S1. List of primary antibodies for immunostaining

Name	Host	Reactivity	Working dilution	Catalog number	Company
MYH7	Rabbit	Mouse, Rabbit, Rat, Human, Zebrafish, etc.	1:200	PA5-69132	Life technologies (Thermo Fisher)
Albumin	Mouse	Rabbit, Human	1:200	GTX102419	GeneTex, Irvine, CA, USA
OTC	Mouse	Rabbit, Human	1:200	GTX64710	GeneTex, Irvine, CA, USA
GS	Mouse	Rabbit, Human	1:200	GTX63065	GeneTex, Irvine, CA, USA

Supplementary Table S2. List of secondary antibodies for immunostaining

Name	Host	Reactivity	Working dilution	Catalog number	Company
Alexa Fluor 488	Goat	Rabbit	1:200	A11034	Invitrogen
Alexa Fluor plus 647	Goat	Mouse	1:200	A32728	Invitrogen

Supplementary Table S3. List of fluorescent stains for fluorescence staining

Name	Working dilution	Catalog number	Company
Rhodamine phalloidin	1:40	R415	Life technologies (Thermo Fisher)
DAPI	1:1000	D1306	Life technologies (Thermo Fisher)
AdipoRedTM Assay Reagent	1:40	PT-7009	Lonza
Hoechst	1:1000	62249	Life technologies (Thermo Fisher)
LIVE/DEAD™ Viability/Cytotoxicity Kit	1:2000	L3224	Life technologies (Thermo Fisher)

Supplementary Table S4. Primer sequences for gene expression analysis

Gene name	Forward primer sequence	Reverse primer sequence
GAPDH	CACCCACTCCTCCACCTTT	TGCTGTAGCCAAATTCGTTG
Beta-Actin	CTGTCCCTGTATGCCTCTG	ATGTCACGCACGATTTCC
HGF	AGTCCAACGGGTCTCAAGTG	CCAAACCACTGCAAAAGGAT
HFN4a	TGCAGGTGTTGACGATGGGCA	ACCACGCACTGCCGGCTAAAT

Gene name	TaqMan™ Gene Expression Assay (FAM) ID
Beta-Actin	Mm02619580_g1
MyoG	Mm00446194_m1
Tnnt1	Mm00449089_m1
Foxo3	Mm01185722_m1
Fbx32	Mm00499523_m1
